# Down-regulation of long non-coding RNA HOTAIR sensitizes breast cancer to trastuzumab

**DOI:** 10.1038/s41598-019-53699-w

**Published:** 2019-12-27

**Authors:** Tianwen Chen, Zeming Liu, Wen Zeng, Tao Huang

**Affiliations:** 1 Department of Breast and Thyroid Surgery, Nanshan Hospital affiliated to Shenzhen University, Shenzhen, China; 2 Department of Breast and Thyroid Surgery, Union Hospital, Tongji Medical College, Huazhong University of Science and Technology, Wuhan, China; 3 Department of Ophthalmology, Zhongnan Hospital, Wuhan University, Wuhan, Hubei China

**Keywords:** Bone cancer, Breast cancer

## Abstract

This study aimed to investigate the roles and possible molecular mechanisms of long non-coding RNA HOTAIR in regulating resistance to trastuzumab in breast cancer. Trastuzumab-resistant breast cancer cell line SK-BR-3-TR was assayed for the expression of HOX antisense intergenic RNA (HOTAIR), epithelial-mesenchymal transition (EMT)-related proteins or genes. Methylation levels of TGF- β, PTEN and cyclin-dependent kinase inhibitor 1B (or P27) were determined. In trastuzumab-resistant cell line, the mRNA level of HOTAIR was significantly up-regulated; in addition, the expression of TGF-β, Snail and Vimentin was also up-regulated, E-cadherin was down-regulated while the expression of HER2, PI3K, AKT, mTOR and MAPK in the HER2 receptor pathway and phosphorylation level of HER2 receptor remained unchanged, the methylation levels of the PTEN gene and TGF-β were increased and decreased, respectively. RNA interference downregulated the HOTAIR level and sensitized the cells to trastuzumab. It also resulted in down-regulation of TGF-β, Snail, Vimentin, p-AKT, p-APK and CyclinD1 and up-regulation of E-cadherin, PTEN and P27. Besides, the methylation levels of the PTEN gene and TGF-β were reduced and increased, respectively. Mouse models grafted with SK-BR-3-TR grew faster than with SK-BR-3-TS and siHOTAIR-SK-BR-3-TR.

## Introduction

Breast cancer is a heterogeneous disease and subtype of HER2-positive breast cancer accounts for about 20–25% of all breast cancer. This subtype is highly proliferative, invasive and easy to transfer distantly. Trastuzumab is a humanized monoclonal antibody against HER2 receptor. It binds the HER2 receptor to suppress the formation of HER2 dimer to deactivate the downstream signaling pathways and inhibit the proliferation of tumor cells and promote the apoptosis of tumor cells^1^. Resistance to trastuzumab is one of the important factors leading to the failure of the treatment of HER2-positive breast cancer. Therefore, identification of new potential targets that overcome the resistance is one of important strategies to improve the survival of patients with HER2-positive breast cancer^2^. In breast cancer resistant to trastuzumab, it might be possible that there are regulatory factors in the upstream of signal pathways responsible for the resistance and the resistance might be derived from the change in these factors that result in abnormal expression of relevant signal pathways and effectors.

Long non-coding RNA (lncRNA) is a class of RNA molecules with a length of more than 200 nucleotides that do not code for proteins but have regulatory roles such as epigenetic regulation. *In vitro* and whole genome studies have shown that HOX antisense intergenic **RNA** (HOTAIR) recruits PRC2 and LSD1 complexes to the promoter regions of target genes in breast cancer, resulting in changed expression of over 850 genes in TGF-β, JAK/STAT, PI3K/AKT and PTEN pathways and increased invasion and metastasis of breast cancer cells^3^. Studies also suggest that HOTAIR is involved in the epithelial to mesenchymal transition (EMT) in breast cancer and associated with maintenance of stemness of breast cancer cells^4–7^. Li *et al*. found that in human laryngeal squamous cell carcinoma model HOTAIR induces the methylation and down-regulation of PTEN, leading to increased PI3K/AKT pathway activity and proliferation and metastasis of cancer cells^8^. However, it is still unclear whether there is a similar regulatory mechanism in breast cancer.

HOTAIR is highly expressed in ER-positive, tamoxifen- resistant breast cancer cell line and in tissue samples from recurrent patients failed to tamoxifen treatment^9^. In addition, the gene is also highly expressed in tamoxifen-resistant cell breast cancer cells deprived of estrogen and the resistance is derived from up-regulation of ER receptor. However, the roles of HOTAIR in resistance to anthracycline, taxane chemotherapy and trastuzumab have not been reported.

In our early study, we obtained stable trastuzumab-resistant cell line SK-BR-3-TR from HER2-positive, trastuzumab-sensitive breast cancer cell line SK-BR-3. Analysis showed that in the resistant cells, the expression of HOTAIR and activity of the PI3K/AKT pathway were up-regulated while the level of PTEN was lowered. We also found that knockdown of HOTAIR sensitized the cells to trastuzumab. Based on these findings, we speculated that once acquired resistance to trastuzumab, HOTAIR is up-regulated, resulting in the methylation of PTEN gene as a result of epigenetic regulation and increased PI3K/AKT pathway activity and subsequently resistance to trastuzumab. To better understand the molecular mechanism of trastuzumab resistance with regarding to HOTAIR, we investigated the expression and activity of genes in the HER2 receptor pathway and EMT- related TGF-β pathway. These findings would provide clues to new treatment targets and strategies.

## Materials and Methods

### Cell line and animals

SK-BR-3 cell line was obtained from ATCC (Manassas, VA) and maintained in RPMI-1640 medium (Gibco, USA) with 10% fetal bovine serum (FBS). Four-week-old specific-pathogen-free, female BALB/c mice weighing 23–27 g (Experimental Animal Center, Huazhong University of Science and Technology, Wuhan, China) were used in this study and were housed under pathogen-free conditions and had access to standard rodent food and water ad libitum. This study received approval by the Animal Research Ethics Board at Shenzhen University and all experiments were performed in accordance with relevant guidelines and regulations.

### Preparation of trastuzumab resistant cells

Trastuzumab sensitive cell line SK-BR-3 was obtained from ATCC (Manassas, VA, USA) and maintained in RPMI-1640 medium (Gibco, USA) with 10% fetal bovine serum (FBS). Cells in the logarithmic growth phase were used to establish trastuzumab resistant cell line. The cells were first grown in complete medium containing 0.5 μg/ml trastuzumab (Roche, Shanghai, 10 times of 50% inhibition concentration), and then grown and subcultured in increasing trastuzumab concentrations from 0.5, 1, 2, 4, 6 to 8 μg/ml. The cells that stably grew in medium containing 8 μg/ml trastuzumab for one month was designated SK-BR-3-TR (trastuzumab-resistant cell line), and maintained in medium containing 4 μg/ml trastuzumab for subsequent experiments.

### Transfection and selection of stably transfected cells

Construction and transfection lentiviral vectors pLenti6/V5 (Invitrogen, USA) harboring siRNAs siHOTAIR-1(5′-GAACGGGAGUACAGAGAGAUU), scramble siHOTAIR (5′-CCACAUGAACGCCCAGAGAUU) and selection of stably transfected cell line HOTAIR-SK-BR-3-TR were performed at Hongboyuan Biotech, Shenzhen, China.

### RT-PCR

Total RNA was extracted by using TRIzol Reagent (Life Technologies, Carlsbad, CA, USA) according to the manufacturer’s protocol. RNA quantity was measured by a SmartSpec Plus spectrophotometer (Bio-Rad, Hercules, CA, USA). RNA purity was evaluated by the A260/A280 ratio. The first strand of cDNA was synthesized using the RNA as template using a cDNA synthesis kit (Cwbiotech, Beijing, China) and quantified using a quantitative fluorescent PCR kit (Cwbiotech, Beijing, China) according to manufacturer’s instructions in 25 µl reactions containing 1 µl cDNA, 1 µl each of primers, 12.5 µl 2x Ultasybr mixture and 9.5 µl ddH_2_O. The cycling conditions were 95 °C for 5 min, followed by 40 cycles, each one consisting of 45 s at 95 °C and 45 s at 60 °C, with a final extension at 72 °C for 45 s. Samples were run in triplicate and the mean value was calculated for each case. The data were managed according to previously described protocol^10^.

### Western blot analysis

Cells were lysed using RIPA buffer (50 mM Tris, pH 7.2; 150 mM NaCl; 1% Triton X-100; and 0.1% SDS) containing protease (1:100, Roche, USA) and phosphatase (1:100, Sigma-Aldrich, USA) inhibitors. The protein concentrations were determined using a bicinchoninic acid assay (Pierce, Thermo Scientific, USA). Sixty μg of the proteins were separated by SDS-PAGE and transferred (Bio-Rad, USA) to PVDF membranes (Millipore, USA). Protein expression levels were quantified using rabbit polyclonal antibodies specific for each protein (TGF-β, Snail and E-cadherin, Abcam, USA, PTEN and Ki67, Sigma, USA). The expression levels of these proteins were standardized to human α-actin using a mouse polyclonal anti-α-actin antibody (Millipore, USA). Primary antibodies were detected using goat anti-rabbit or goat anti-mouse horseradish peroxidase (HRP)-conjugated secondary antibodies (Santa Cruz Biotechnology, US A). Immunoreactive bands were visualized using Western Lighting Chemiluminescence Reagent Plus (PerkinElmer, USA) according to the manufacturer’s instructions, and then quantified by densitometry using a ChemiGenius Gel Bio Imaging System (Syngene, USA).

### Flow cytometry

Flow cytometry was used to detect apoptosis. Cells were collected, washed once with PBS and suspended in PBS. 10^6^ re-suspended cells were added to 100 µl binding buffer and labeled with Annexin V and propidiumiodide (PI) following the manufacturer’s instruction (Biosea Biotechnology, Beijing, China). Flow cytometer (Bection Dikinson, USA) was used to assess the apoptotic cells. The quantitation of apoptotic cells was calculated by CellQuest software.

### Transwell invasion assay

For the assessment of invasion, transfected cells were cultured for 24 h and 2 × 10^4^ transfected cells in serum-free medium were placed into the upper chamber of an insert coated with Matrigel (BD Bioscience, USA). Media containing 20% FBS were added to the lower chambers. After 24 h of incubation, the cells remaining on the upper membrane were removed with cotton wool, whereas the cells that had invaded through the membrane were stained with 2% crystal violet in 25% methanol/PBS for 20 min, washed three times with PBS, imaged and counted in five fields using an EVOS XL Core inverted microscope (Life Technologies, USA). The experiments were independently repeated three times.

### Methylation-specific PCR

Methylation-specific PCR was preformed as described previously^11^. Briefly, DNA was isolated from cells using DNeasy Blood & Tissue kit (Qiagen, Inc.). The extracted DNA was then subjected to bisulfite treatment using the EpiTect Bisulfite kit (Qiagen, Inc.) according to the manufacturer’s instructions. The amplification reaction was conducted under the following conditions: 95 °C for 5 min. followed by 30 cycles of 94 °C for 30 sec, 54 °C for 30 sec, 72 °C for 60 sec, with a final extension of 10 min at 72 °C. The PCR products were visualized on 2% agarose gels.

### Mouse experiments

The nude mice were randomly divided into three groups, each consisting of 6 animal. 0.2 ml cell suspension (10^7^ cells) were injected into subcutaneously into the back of nude mice after surface sterilization. The first group was injected with SK-BR-3-TS cells while the other two groups were injected with SK-BR-3-TR and HOTAIR knockdown cells. When the tumors were between 800 and 1000 mm^3^, the mice were injected with trastuzumab (8 mg/kg) every week for four weeks via the tail veins. The mice were observed daily for activity, eating, urine, skin color and measured for the size of the tumors once appeared using vernier caliper. The animals were weighted weekly. On day 35, the mice were sacrificed by cervical dislocation and the tumors were isolated for analysis.

### Statistical analysis

All data were expressed as means ± standard derivation (SD). Statistical comparisons between groups were assessed by using the Student’s *t*-test. *P* < 0.05 was considered statistically significant.

## Results

### Induction of trastuzumab resistance and establishment of resistant cell line SK-BR-3-TR

**Figure 1:**
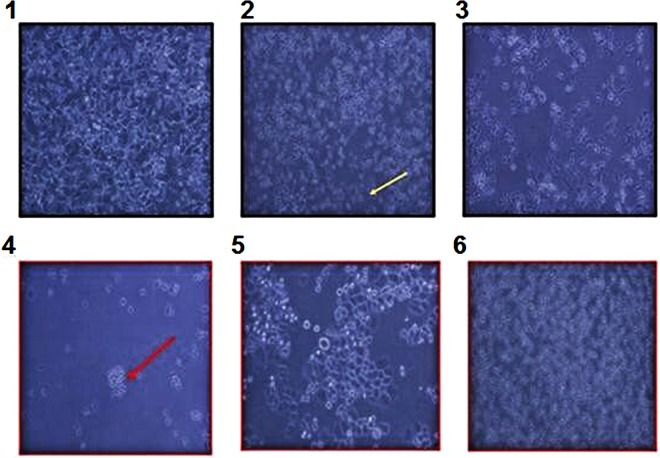
Induction, screening and selection of trastuzumab-resistant cells. 1. cells before exposed to trastuzumab, 2 and 3, cells exposed to trastuzumab, showing apoptosis, 4 and 5, resistant colony and proliferation of resistant cells, and 6, stable resistant cells growing in medium containing 8 μg/ml trastuzumab. Red arrow indicates resistant cell and colony and yellow arrows indicates apoptotic cell and colony.

Trastuzumab-sensitive cells were induced to form colonies resistant to trastuzumab at 0.5 μg/ml and then cultured in increasing concentration of trastuzumab of up to 8 μg/ml (Fig. 1). After several rounds of culture and selection, a stable trastuzumab resistant cell line SK-BR-3-TR was obtained (Fig. 1), which could grew stably in medium containing 8 μg/ml trastuzumab which was over 10 times the level that trastuzumab-sensitive cells can tolerate.

### Expression of HOTAIR following knockdown

**Figure 2:**
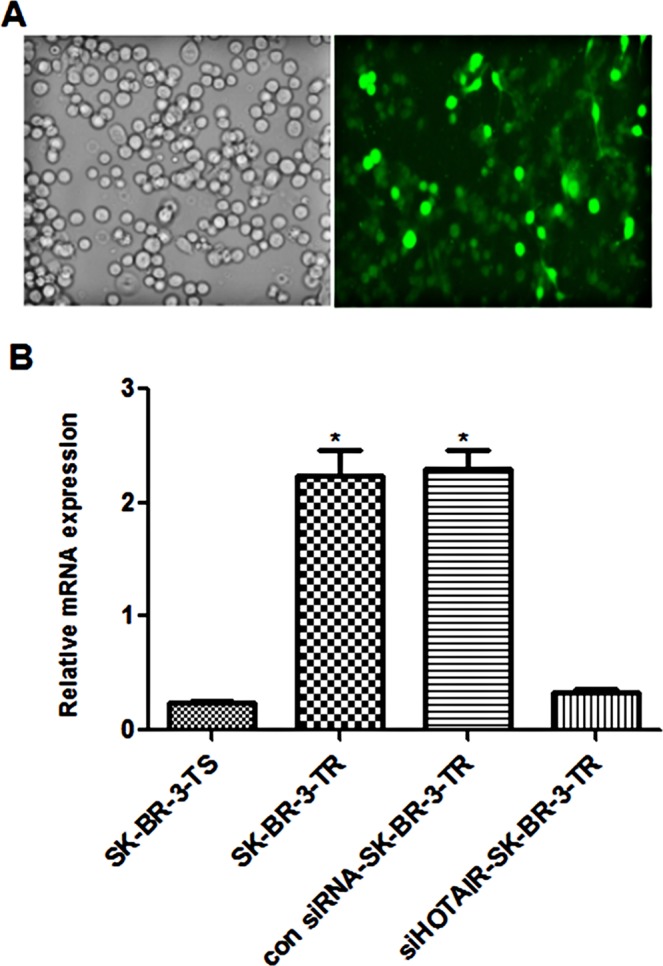
Expression of GFP and HOTAIR in of trastuzumab resistant cells following transfection with HOTAIR knockdown lentivirus. (**A**) Microphoto of cells under inverted phase contrast microscope and fluorescence microscope; (**B**) mRNA level of HOTAIR in trastuzumab- resistant and sensitive cells. *P < 0.05 vs control.

The lentivirus vector used for HOTAIR knockdown contained green fluorescent protein (GFP). To assess the transfection efficiency, we examined the GFP expression. 48 h after transfection, GFP was observed in 70–80% cells (Fig. 2A). Since the expression level of HOTAIR has not be comparatively examined in trastuzumab-resistant cells, we first compared its level in the sensitive and resistant cells. RT-PCR analysis showed that the mRNA level of HOTAIR was up-regulated in the resistant cells as compared with the sensitive cells (2.216 ± 0.332 vs 0.326 ± 0.05, *P* = 0.0006, Fig. 2B), indicating that HOTAIR might play a role in trastuzumab resistance. Once the resistant cells were transfected with the knockdown virus, the level of HOTAIR was reduced by 80% as compared with the cells transfected with control siRNA (0.473 ± 0.146 vs 2.108 ± 0.503, *P* < 0.05) (Fig. 2B).

### Proliferation, cell cycle and apoptosis following HOTAIR knockdown

**Figure 3:**
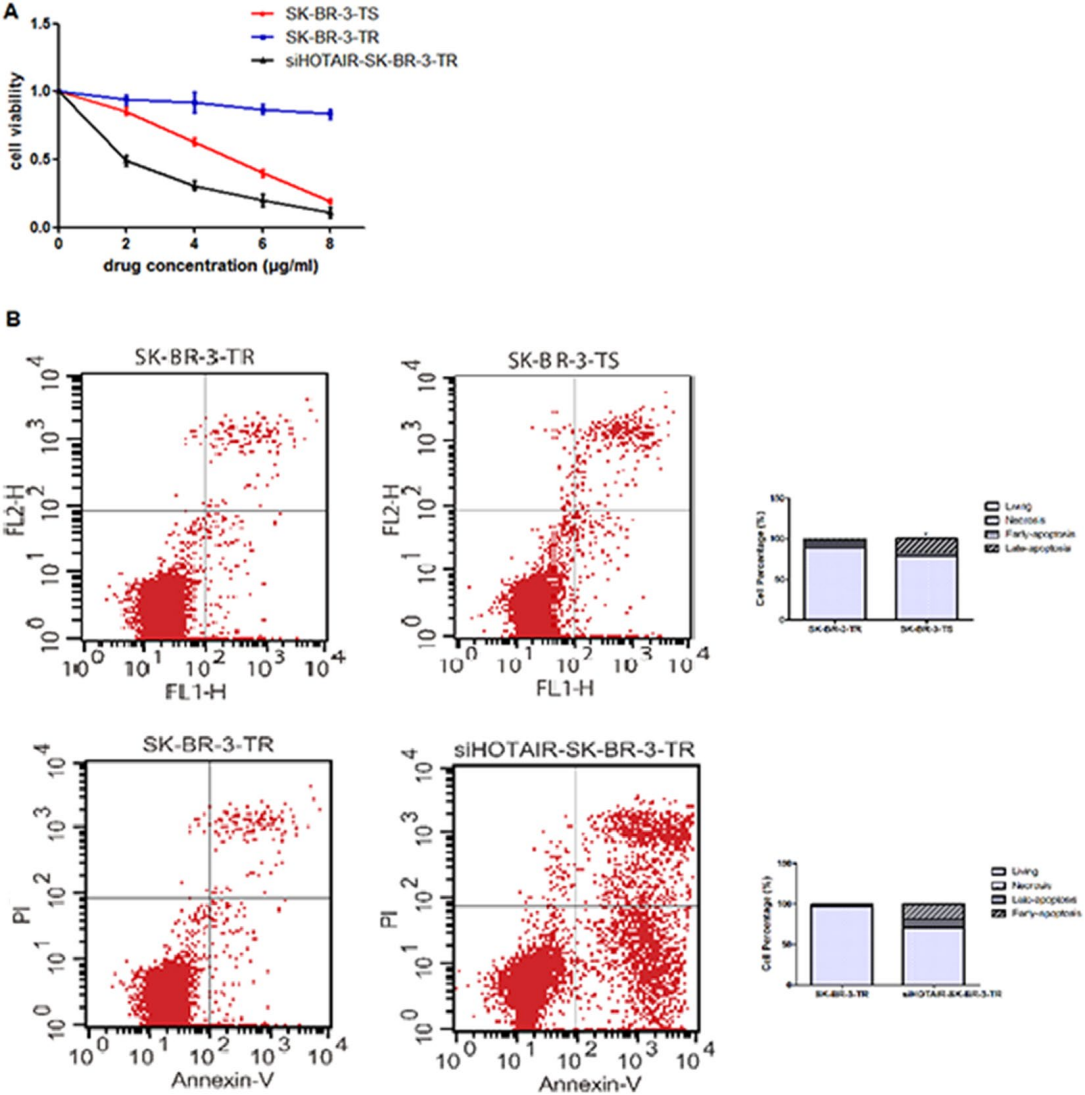
Relative viability and apoptosis of breast cancer cells treated with trastuzumab and following HOTAIR knockdown. (**A**) Cell viability; (**B**) apoptosis.

The MTT assays showed that the relative viability of sensitive cells treated with 4 μg/ml trastuzumab for 24 h was reduced by 40% as compared with untreated cells (1 vs 0.62 ± 0.02) with a IC50 of 0.25 μg/ml, while that of resistant cells remained unchanged (1 vs 0.87 ± 0.03), even at a trastuzumab concentration of 8 μg/ml (1 vs 0.83 ± 0.03, Table 1)). On contrast, the viability of sensitive cells were further reduced (1 vs 0.23 ± 0.02) at the trastuzumab concentration (Fig. 3A). After HOTAIR knockdown, which results in 80% reduction in HOTAIR level, the sensitivity to trastuzumab was increased in the resistant cells with a IC50 of 0. 0.16 μg/ml, which loss their viability as the concentration of trastuzumab increased (Fig. 3A). The sensitivity of these knockdown cells was even greater than the sensitive cells (Fig. 3A). These findings further suggested that HOTAIR is involved in the trastuzumab resistance.

**Table 1:** Size (mm^3^) of tumors before trastuzumab intervention.

Days of observation	siHOTAIR-SK-BR-3-TR	SK-BR-3-TR	SK-BR-3-TS
3	42.16 ± 5.26	40.28 ± 4.58	45.65 ± 4.85
6	282.35 ± 20.31	358.36 ± 14.25	302.68 ± 25.46
9	414.50 ± 18.57	626.66 ± 18.02	492.08 ± 26.31
12	764.94 ± 86.54	1137.64 ± 75.50	793.66 ± 78.04
14	851.55 ± 45.22	1365.87 ± 145.20	880.60 ± 25.50
21	759.25 ± 24.44	1556.21 ± 156.54	790.65 ± 18.60
28	625.20 ± 46.78	1794.08 ± 88.60	675.00 ± 54.32
35	576.31 ± 68.22	2088.60 ± 184.65	540.40 ± 92.63
42	462.55 ± 65.37	2230.20 ± 384.65	480.40 ± 102.63

**Figure 4:**
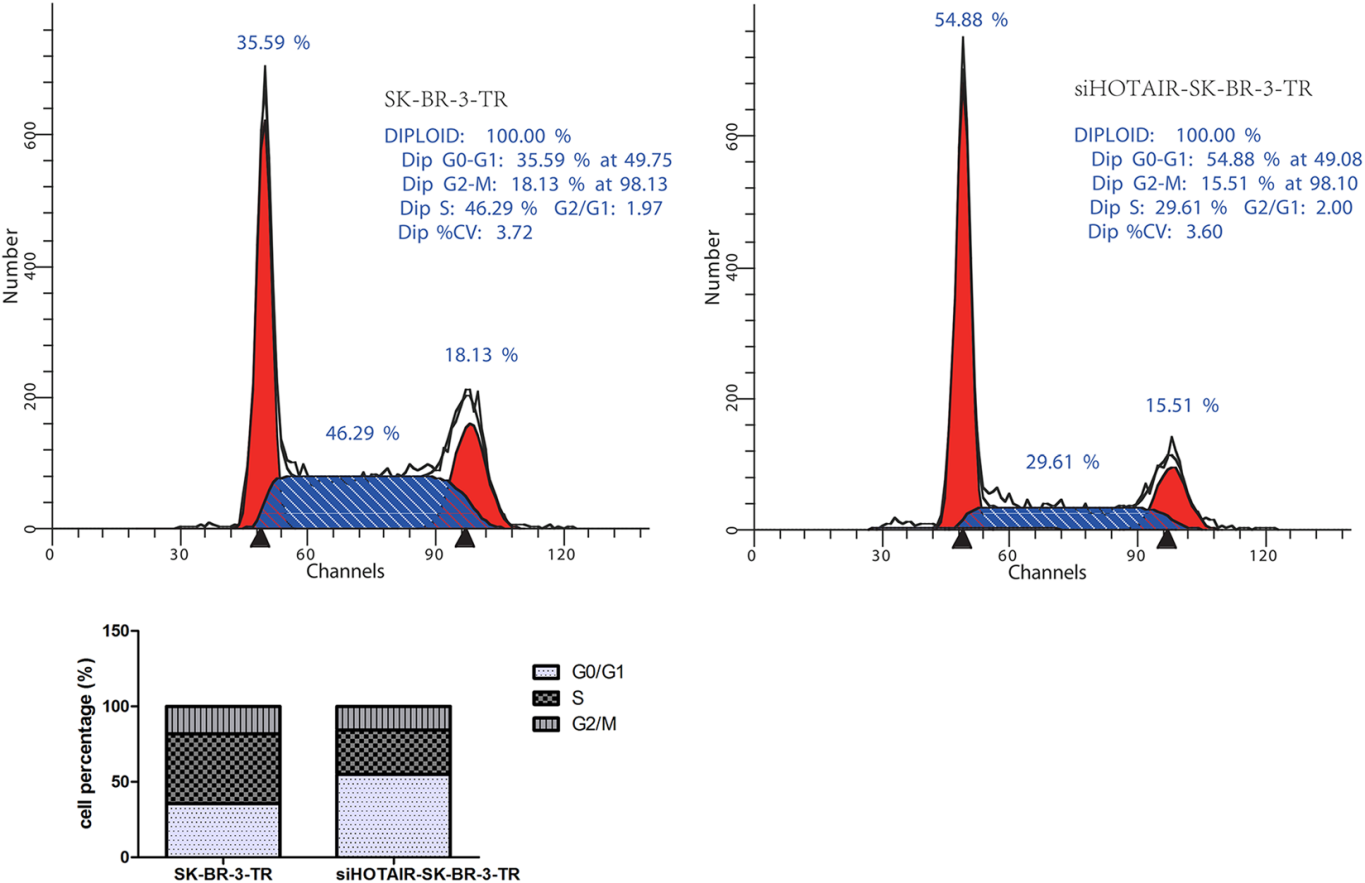
Cell cycle assessments of breast cancer cells treated with trastuzumab and following HOTAIR knockdown.

In order to better understand the reduced viability, we examined apoptosis in these cells. As shown in Fig. 3B, after 96 hours of incubation with trastuzumab, the percentage of apoptotic cells, particularly late apoptotic cells, were significantly less in the resistant cells as compared with the sensitive cells (3.51 ± 1.70 vs 17.80 ± 5.38, *P* = 0.032), suggesting that the higher proliferative ability in the resistant cells might be partially attributed to their increased anti-apoptotic ability. Because cell proliferation and apoptosis are often related to cell cycle, we examined the distribution of cells in different cell cycle following HOTAIR knockdown. Near 46% of the cells were in S phase before the knockdown, while the level reduced to 15% once HOTAIR was knockdown. On the other hand, the proportion of cells in G0/G1 phase increased (Fig. 4), suggesting that HOTAIR knockdown arrests the cells at G0/G1 in the resistant cell line. Furthermore, the proportion of apoptotic cells, particularly early cells, was increased after HOTAIR knockdown in SK-BR-3-TR cells (Fig. 4), suggesting that HOTAIR is involved in the regulation of cell cycle and apoptosis, and subsequently proliferation in SK-BR-3-TR cell.

### Invasion and expression of ETM-related genes following HOTAIR knockdown

**Figure 5:**
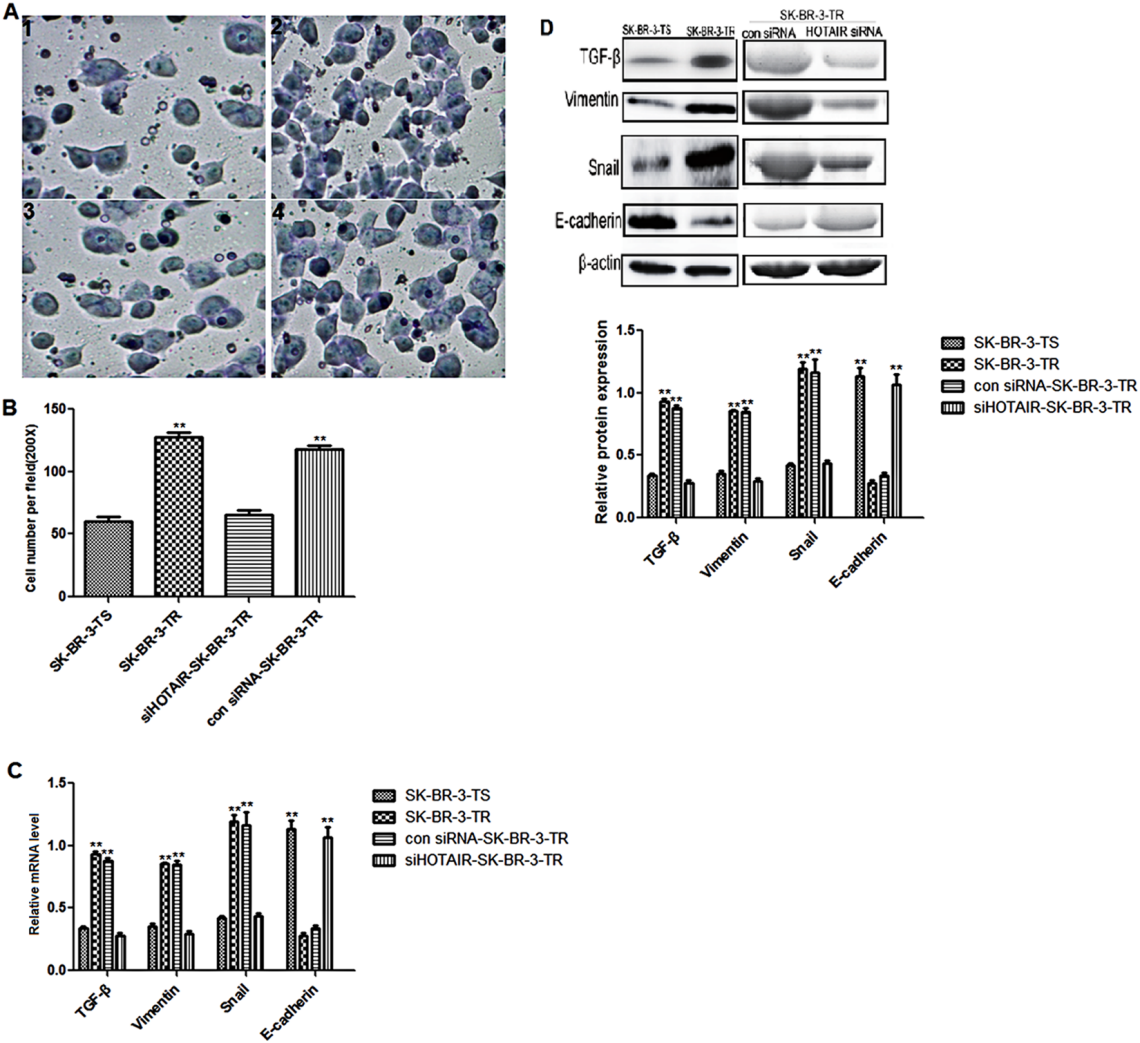
Transwell assay and expression of ETM-related genes in trastuzumab sensitive and resistant cells following HOTAIR knockdown. (**A**) 1–2, microphotos of trastuzumab sensitive and resistant cells in Transwell assay, 3–4, microphotos of trastuzumab resistant cells in Transwell assay after transfected with siHOTAIR and control siRNA (**B**) number of migrated cells; (**C**) mRNA levels of ETM-related genes; (**D**) upper panel, representative Western blots and lower panel: protein levels of ETM-related genes. **P < 0.01 vs control.

Compared with the sensitive cells, the resistant cells had higher invasion ability as revealed in Transwell assay (Fig. 5A, 1–2, and B, Supplementary Figure 5). After HOTAIR knockdown, the percentage of SK-BR-3-TR cells migrated through the upper chamber in the Transwell assay was significantly reduced (Fig. 5A, 3–4, and B), suggesting that the up-regulation HOTAIR in the resistant cells might be responsible for the increased invasion ability in SK-BR-3-TR cells.

We then examined the expression of ETM-related genes. It was found that TGF-β, Vimentin and Snail were downregulated and E-cadherin upregulated significantly following HOTAIR knockdown (Fig. 5C) at mRNA and protein levels (Fig. 5D).

### Activity of PI3K/AKT/mTOR and MEK/MAPK signaling pathways

**Figure 6:**
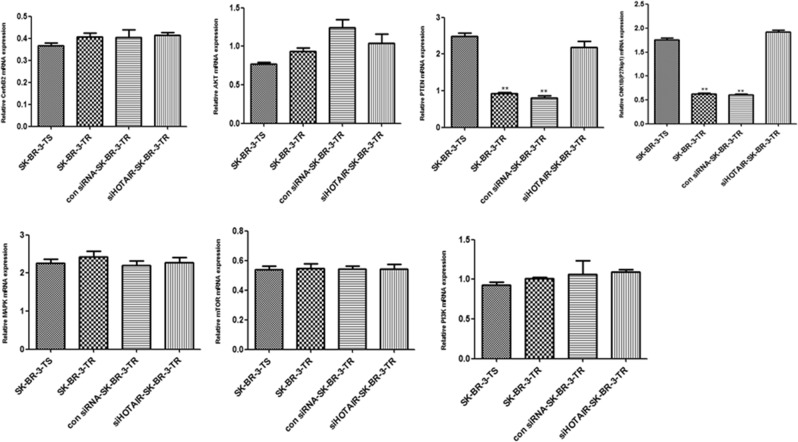
mRNA levels of genes in the PI3K/AKT/mTOR, MEK/MAPK and HER2 receptor pathways following SHOTAIR knockdown. **P < 0.01 vs control.

Compared with the sensitive cells, the mRNA levels of CerbB2, PI3K, AKT and the in the HER2 receptor pathway and mTOR in the MAPK pathway were not changed (*P* > 0.05) in the resistant cells, but PTEN and P27 were significantly down-regulated. Compared with SK-BR-TR cells transfected with Con siRNA, HOTAlR knockdown did not change the mRNA levels of CerbB2, PI3K, AKT, MAPK and mTOR but significantly up-regulated the expression of PTEN and P27 (*P* < 0.05, Fig. 6).

**Figure 7:**
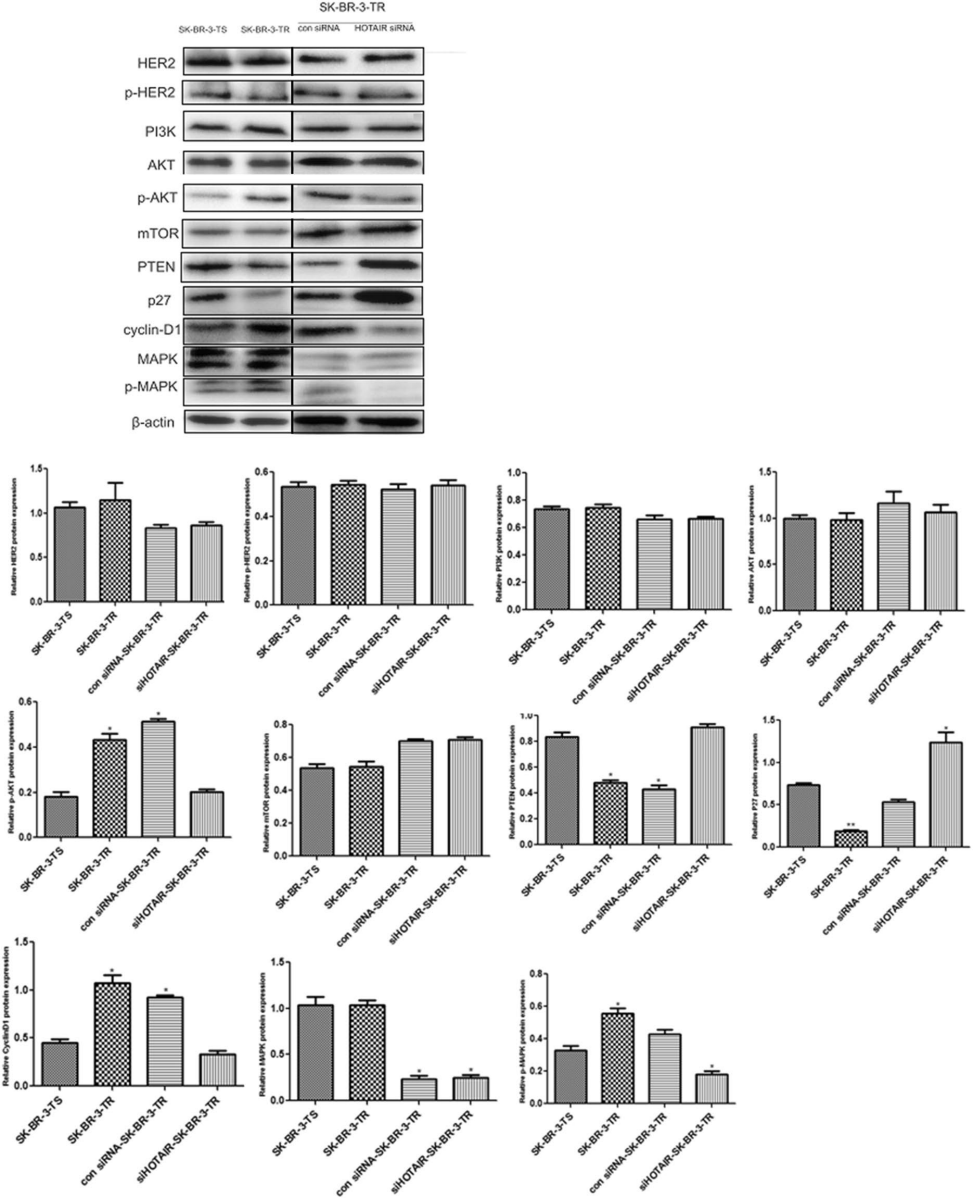
Protein levels of genes in the PI3K/AKT/mTOR, MEK/MAPK and HER2 receptor pathways following SHOTAIR knockdown. Upper panel: representative Western blot, lower panel: protein and phosphorylation levels. **P < 0.01 vs control.

Since the activity of these two pathways is regulated by posttranstriptional phosphorylation, we also measured the expression of these genes at protein level. The results were similar to what happen to their mRNA levels (Fig. 7). Furthermore, the phosphorylation level of AKT, MAPK as well as CyclinD1 was significantly increased. However, the level and phosphorylation level of HER2 remained unchanged in both sensitive and resistant cells (Fig. 7, Supplementary Figure 7). Compared with SK-BR-TR cells transfected with control siRNA, HOTAlR knockdown did not change the protein levels of PI3K, AKT, mTOR and MAPK, but up-regulated these of PTEN and P27, down-regulated the phosphorylation level of AKT, MAPK and CyclinD1. The levels of HER2 and phosphated HER2 (p- HER2) remains similar before and after HOTAlR knockdown (Fig. 7). These findings suggested that HOTAIR does not influence the activity of these pathways via regulating the expression of genes in the signaling nodes but via up-regulating the expression of pathway regulatory genes such as PTEN and P27 and CylinD1 pathway to increase the intrinsic activity.

### Methylation of TGF- β, PTEN and p27

**Figure 8:**
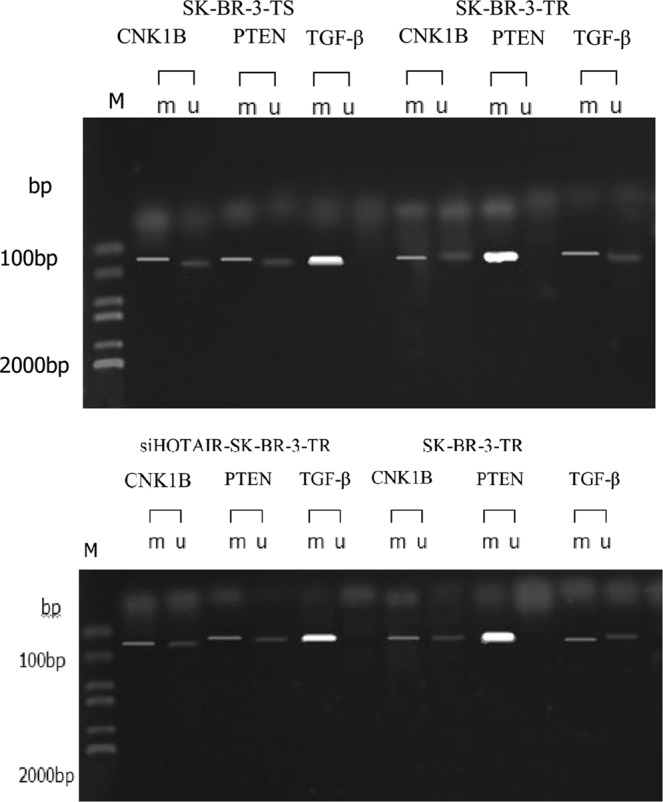
Methylation of P27, PTEN and TGF- β genes in SK-BR-3-TR cells following HOTAIR knockdown.

HOTAIR has bidirectional epigenetic regulatory functions, which activates or deactivates the expression of genes by demethylating or methylating genes, respectively^3,12^. Since the expression levels of TGF- β, PTEN and p27 were changed in the resistant cells, we speculated that HOTAIR might epigenetically modify the genes to different levels of methylation. To test this hypothesis, we examined the methylation levels of these genes. Compared with sensitive cells, the methylation level of the p27 gene was unchanged in the resistant cells, while PTEN and TGF-β were significantly methylated or demethylated (Fig. 8, upper panel). When HOTAIR was knockdown, the methylation levels of PTEN and TGF-β were up-and down-regulated, respectively in the resistant cells as compared with the sensitive cells, while the methylation level of the p27 gene remained unchanged (Fig. 8, lower panel).

### Tumor growth in nude mice

**Figure 9:**
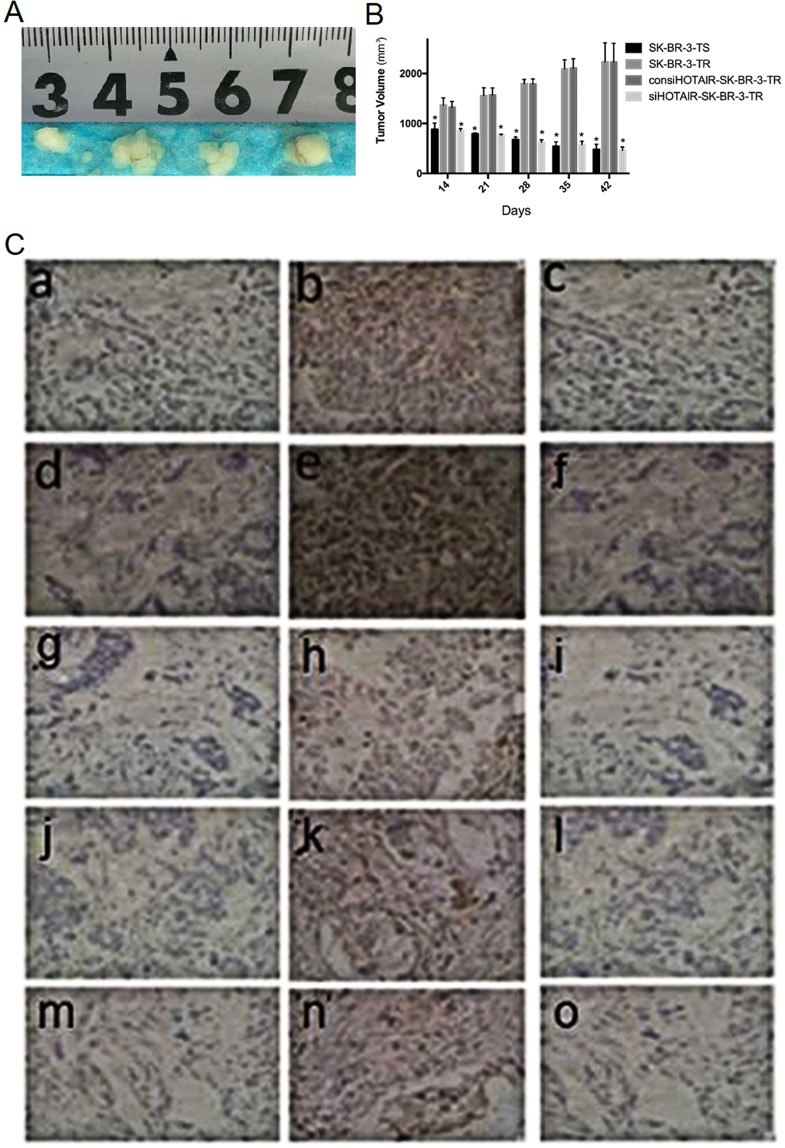
Tumors developed after trastuzumab intervention in mice injected with different breast cells (from left to right, derived from SK-BR-3-TS, SK-BR-3-TR, con siHOTAIR-SK-BR-3-TR and siHOTAIR-SK-BR-3-TR cells) and expression of EMT and HER2 pathway-related proteins in the tumors (IHC, x 200). (**A**) tumor morphology (n = 6), (**B**) average tumor volume, (**C**) immunohistochemical analysis. a and c: Snail−; b: Snail+; d and f: Ki67−; e: Ki67+; g and i: E-cadherin−; h: E-cadherin+; j and l: TGF-β−; k: TGF-β+; m and o: PTEN−; n: PTEN+. *P < 0.05 vs control.

14 days after the injection of tumor cells, the tumor volumes were all more than 800 mm^3^, and SK-BR-3-TR cells produced the largest tumor (Table 1). The tumor derived from SK-BR-3-TR cells continued to grow after intervention with trastuzumab from day 14 and reached 2230.20 mm^3^ at the end of experiments (42 days after the injection), tumors injected with SK-BR-3-TS and siHOTAIR-SK-BR-3-TR ceased to grow (Fig. 9A,B).

Immunohistochemical analysis showed that at the end of experiments, the positive rates of TGF-β and Snail proteins were lower but insignificantly in tumors derived from siHOTAIR-SK-BR-3-TR cells than from SK-BR-3-TR cells (16.7% and 33.3% vs 66.7% and 83.3%, *P = *0.242). The positive rates of E-cadherin and PTENY were higher, but insignificantly in tumors derived from siHOTAIR-SK-BR-3-TR cells than from SK-BR-3-TR cells (*P* = 0.242), while these of Ki67 were the same (83.3%, *P = *1.000) (Fig. 9C, Table 2).

**Table 2:** Positive rates of relevant proteins in tumor tissues derived from different cell lines (n = 6).

Marker	siHOTAIR-SK-BR-3-TR		SK-BR-3-TR		SK-BR-3-TS	
	Negative	Positive	Negative	Positive	Negative	Positive
TGF-β	5	1	2	4	4	2
Snail	4	2	1	5	4	2
E-cadherin	2	4	5	1	2	4
PTEN	1	5	5	1	2	4
Ki67	1	5	1	5	2	4

## Discussion

Primary and acquired resistance to trastuzumab has been a challenge to breast cancer-targeted therapy^2^. Since preclinical trial and approval of trastuzumab for clinical use, the mechanisms underlying the trastuzumab resistance has been intensively studied. For such studies, it is necessary to establish a cancer model with acquired trastuzumab resistance. Resistant cells were successfully obtained by exposing the cells to high concentration of trastuzumab for 2–3 months^9,13^. In our study, we modified the exposing method and obtained the stable resistant cells in about 7 months month.

The mechanisms underlying trastuzumab resistance has not been fully elucidated, although increased activity of EMT and PI3K/AKT/mTOR signaling pathways has been considered the leading factors^14,15^. Shi *et al*. found that lncRNA ATB increases the activity of TGF-β pathway in SK-BR-3 cells and promotes EMT to induce trastuzumab resistance, which also results in increased invasion and metastasis^16^. However, how HOTAIR is involved in trastuzumab resistance in breast cancer has not been reported.

Our *in vitro* experimental results showed that in SK-BR-3-TR cells, HOTAIR was significantly up-regulated as compared with the parental cells, resulting in increased proliferation and invasion ability, and reduced apoptosis. When HOTAIR was knockdown by lentivirus-mediated RNA interference, the proliferation and invasion ability was reduced and early and late apoptosis increased. Previous study showed that HOTAIR promotes the invasion and metastasis of tumor cells and inhibits the apoptosis of tumor cells^3^. Our *in vivo* experiments further showed that the tumor derived from siHOTAIR-SK-BR-3-TR cells grew significantly slower than the tumor derived from SK-BR-3-TR cells. These findings suggest that HOTAIR is involved in acquired resistance to trastuzumab in SK-BR-3-TR cells.

To better understand the molecular mechanisms underlying the trastuzumab resistance mediated by high expression of HOTAIR, we compared the intrinsic activity of HER2 receptor signaling pathway-related PI3K/AKT/mTOR and MEK/MAPK pathways in the sensitive and resistant cells. The results showed that once resistance to trastuzumab was acquired, the transcription and translation of CerbB2, PIK3CA, AKT, mTOR and MAPK as well as the phosphorylation of HER2 receptor were not affected significantly. However, the levels of p-AKT, p-MAPK and CyclinD1 were up-regulated and PTEN and P27 down-regulated. Moreover, the methylation of PTEN, but not p27 in SK-BR-3-TR cells was increased. Knockdown of HOTAIR resulted in down-regulation of p-AKT, p-MAPK and CyclinD1, and up-regulation of PTEN and P27, and unchanged methylation of p27. These data suggest that HOTAIR does not activate the pathway activity by up-regulating the expression of pathway-related molecules, but by epigenetic methylation of important pathway regulator PTEN, leading to its reduced transcription and translation.

In addition, we also examined the intrinsic activity of TGF-β, Snail, E-cadherin and Vimentin in the EMT-related signal pathway. The results showed that in the resistant cells, the transcription and translation of TGF-β, Snail and Vimentin were up-regulated while E-cadherin was down-regulated. After siRNA interfering of HOTAIR, the results were reversed. Methylation analysis showed that the methylation of TGF-β was reduced in the resistant cells. These findings indicate that HOTAIR can epigenetically demethylate TGF-β in the resistant cells to up-regulate TGF-β expression, and subsequently up-regulate the downstream genes Snail and Vimentin and down-regulate E-cadherin to facilitate EMT^12^, which is one of the leading mechanisms underlying trastuzumab resistance in breast cancer^15,17,18^. In addition, TGF-β has been shown to enhance the activity of MEK/MAPK signaling pathway through the “cross-talk” to promote the proliferation and invasion of tumor cells^19^.

Taken together, it is clear that HOTAIR is up-regulated in trastuzumab-resistant cell line SK-BR-3-TR and blocking of HOTAIR expression restores the sensitivity. HOTAIR is involved in acquired resistance via epigenetic modification of methylation in PTEN, demethylation of TGF- β and cross talk effects from up-and down-regulation of TGF- β and PTEN that enhance the HER2 phosphorylation –independent activity of the MEK/MAPK pathway to promote the proliferation and invasion of tumor cells. In addition, HOTAIR also regulates the expression of P27, CylinD1 and CDK4 to promote the transition of tumor cells from G1 phase to S phase to inhibit the apoptosis through non-epigenetic mechanism or other indirect regulatory mechanisms.

However, there are still some limitations in this study. The number of animal samples used was relatively small and the study was done mostly using cell lines. Although *in vivo* studies have demonstrated that mice derived from siHOTAIR-SK-BR-3-TR cells are sensitized to trastuzumab, the smaller sample size and limitations of immunohistochemical analysis were unable to show statistically significant difference in positive rates of HER2 receptor pathway- and TGF-β pathway-related genes between the mice derived from SK-BR-3-TR cells and SK-BR-3-TR cells. The study fails short to demonstrate the *in vivo* relationship between HOTAIR and molecules in the HER2recepor pathways or EMT signal pathway. Due to resource limitation, we was unable to screen lncRNA in the resistant cells to identify other highly expressed lncRNAs and to elucidate if there are interaction between lncRNA or between microRNA and lncRNA that might also be responsible for trastuzumab resistance. It is very desirable to use larger number of HER2-positive breast cancer samples, particularly those from patients that have undergone targeted trastuzumab therapy and have developed acquired resistance for retrospective and prospective analysis. This would allow better validation and understanding of the relationship between HOTAIR and trastuzumab resistance in breast cancer and its molecular mechanism. In addition, more works are needed to elucidate the regulatory mechanisms of HOTAIR itself and on the expression of p27. Although we have preliminarily investigated the methylation of the HOTAIR target genes, more studies are required to examine the methylation or demethylation sites in the promoter regions. These studies would provide better insights on targeted inhibition of HOTAIR and new strategies to reverse the resistance.

## Supplementary information

Supplementary information
