# Supplementary material for: Down-regulation of long non-coding RNA HOTAIR sensitizes breast cancer to trastuzumab
Source: Sci Rep. 2019 Dec 27;9:19881. doi: 10.1038/s41598-019-53699-w (PMC6934784; doi:10.1038/s41598-019-53699-w)
Supplement: Supplementary file 1 — Supplementary information [file 41598_2019_53699_MOESM1_ESM.pdf]

Supplementary Information

Title: **Down-regulation of long non-coding RNA HOTAIR sensitizes breast cancer to trastuzumab**

Title: List of authors: Tianwen Chen[1,$], Zeming Liu[2,$], Wen Zeng[3], Tao Huang[2,*]

[1]Department of Breast and Thyroid Surgery, Nanshan Hospital affiliated to Shenzhen University, Shenzhen, China

[2]Department of Breast and Thyroid Surgery, Union Hospital, Tongji Medical College, Huazhong University of Science and Technology, Wuhan, China

[3]Department of Ophthalmology, Zhongnan Hospital, Wuhan University, Wuhan, Hubei, China

SUPPLEMENTARY FIGURE LEGENDS

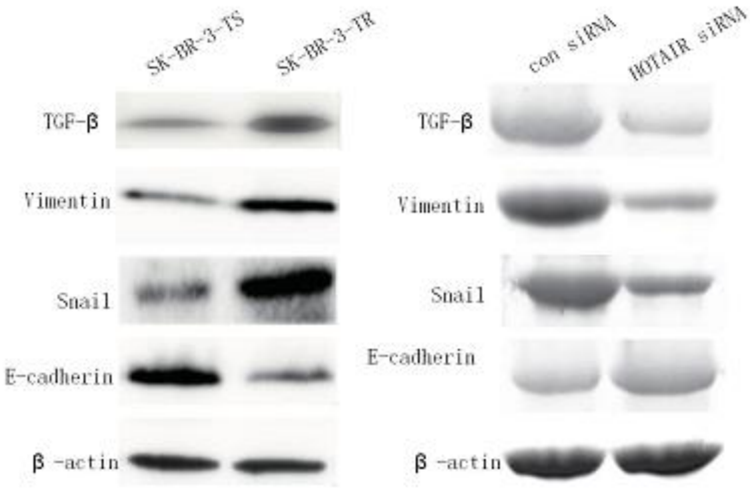

Supplementary Figure 5: Protein blots of of the ETM-related genes following SHOTAIR knockdown. Western blotting was performed according to standard protocols (Biorad Turboblot System). Primary antibodies were detected using goat anti-rabbit or goat anti-mouse horseradish peroxidase (HRP)-conjugated secondary antibodies (Santa Cruz Biotechnology, US A). Immunoreactive bands were visualized using Western Lighting Chemiluminescence Reagent Plus (PerkinElmer, USA).

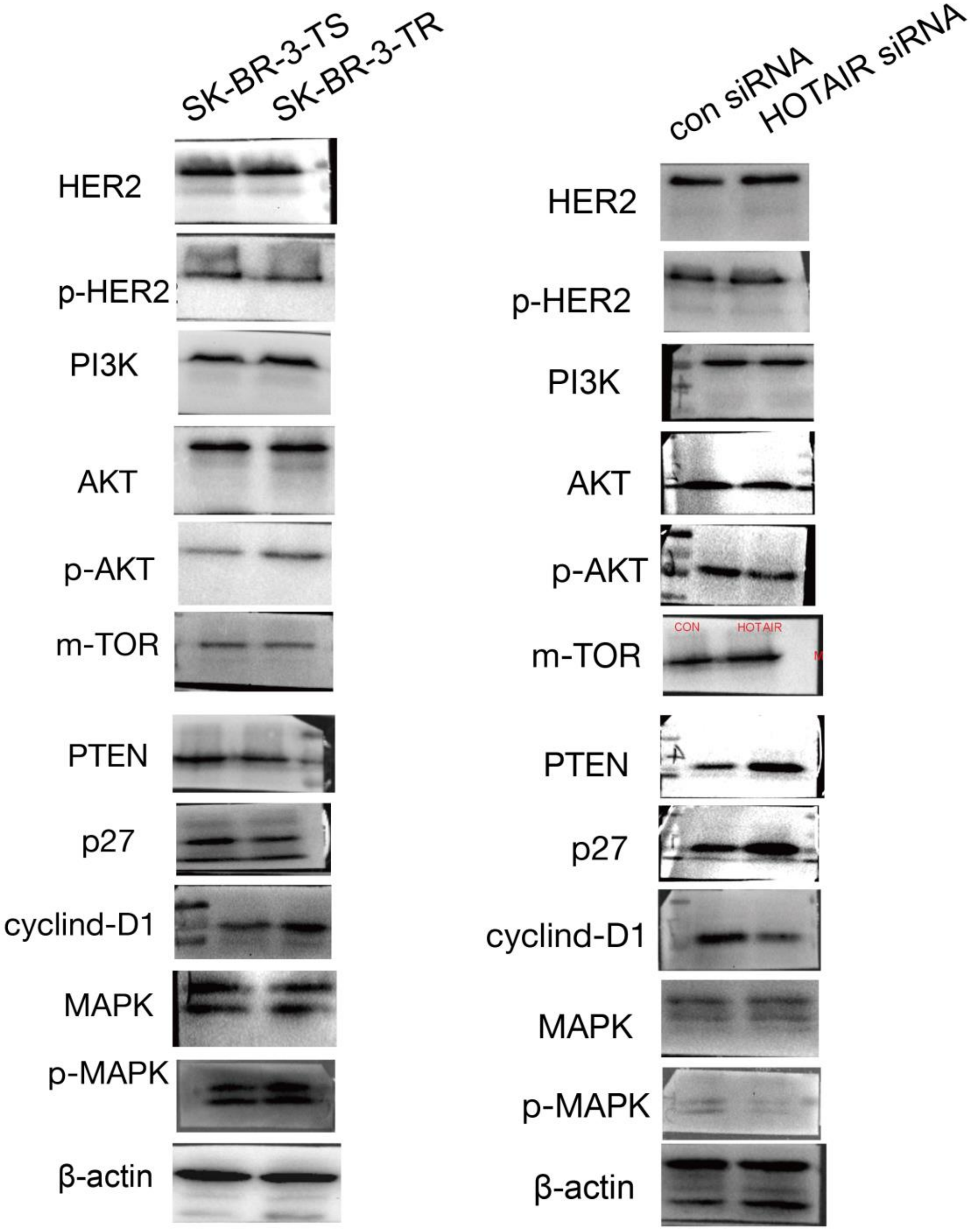
SK-BR-3-TS
SK-BR-3-TR
HER2
p-HER2
PI3K
AKT
p-AKT
m-TOR
PTEN
p27
cyclind-D1
MAPK
p-MAPK
β-actin
con siRNA
HOTAIR siRNA
HER2
p-HER2
PI3K
AKT
p-AKT
CON
HOTAIR
m-TOR
PTEN
p27
cyclind-D1
MAPK
p-MAPK
β-actin

Supplementary Figure 7: Protein levels of genes in the PI3K/AKT/mTOR, MEK/MAPK and HER2 receptor pathways following SHOTAIR knockdown. Western blotting was performed according to standard protocols (Biorad Turboblot System). Primary antibodies were detected using goat anti-rabbit or goat anti-mouse horseradish peroxidase (HRP)-conjugated secondary antibodies (Santa Cruz Biotechnology, US A). Immunoreactive bands were visualized using Western Lighting Chemiluminescence Reagent Plus (PerkinElmer, USA).
